# Construction of Fire-Retardant PEO Composite Based on Calcium Sulfate Whiskers Fabricated from Phosphogypsum and DOPO Derivatives

**DOI:** 10.3390/polym17121588

**Published:** 2025-06-06

**Authors:** Jie Zhang, Wei Yan, Weijiang Huang, Kui Wang, Qin Tian, Chunyun Tu, Xingyu Guan, Shaoyuan Wu, Xuan Ba, Chunle Wei, Tong Ye, Jingyu Chen, Yi Zhang

**Affiliations:** 1 School of Materials Science and Engineering, Guiyang University, Guiyang 550005, China; z2601945295@163.com (J.Z.); huangweijiang_2007@126.com (W.H.); gyxywkui@163.com (K.W.); mabtianqin@126.com (Q.T.); yidapa@sina.cn (C.T.); gxy712@163.com (X.G.); 18785593078@163.com (S.W.); 18185251129@163.com (X.B.); 15772461681@163.com (C.W.); yetong150853@163.com (T.Y.);; 2 National Engineering Research Center for Compounding and Modifcation of Polymer Materials, Guiyang 550014, China

**Keywords:** PEO, flame retardancy, calcium sulfate whiskers, synergistic effect

## Abstract

Incorporating a 9,10-dihydro-9-oxa-10-phosphaphenanthrene-10-oxide-based derivative (1,4-bis(diphenoxyphosphoryl)piperazine, DIDOPO) in combination with modified calcium sulfate whiskers (MCSWs) improved the flame retardancy, thermal stability, and rheological properties of a polyethylene oxide (PEO) composite. The synergistic flame-retardant effect of DIDOPO and MCSW on the PEO system was investigated. After introducing 5 wt.% MCSW and 10 wt.% DIDOPO into PEO, the UL-94 rating of the composite reached V-0, and the limiting oxygen index was increased to 26.5%. Additionally, the peak and average heat release rates and total heat release of the PEO/10% DIDOPO/5% MCSW composite decreased by 38.9%, 22%, and 20.07%, respectively. The results of a thermogravimetric analysis (TGA) revealed that PEO/10% DIDOPO/5% MCSW displayed an improved initial thermal stability and rate of char formation compared to those of the PEO matrix. The results of TGA/Fourier transform infrared analysis indicated that the composites exhibited phosphorus-containing groups during thermal degradation, based on the characteristic absorption peaks, and increased amounts of gas-phase volatiles. The morphologies and structures of the residues indicated that the PEO/10% DIDOPO/5% MCSW blend was less stable than PEO during combustion. The MCSW mixture formed a denser, more continuous carbon layer on the composite surface during combustion. The rheological behavior indicated that the high complex viscosity and moduli of PEO/10% DIDOPO/5% MCSW promoted the cross-linking network structure of the condensed phase during combustion. MCSW exhibited an excellent flame retardancy and improved thermal stability, which are potentially promising for use in fire safety applications.

## 1. Introduction

Polyethylene oxide (PEO) is widely used in solid-state batteries, flexible electronics, and biomedicine owing to its excellent film-forming properties, ionic conductivity, and biocompatibility [1,2,3]. However, the hydrocarbon structure of its molecular chain leads to a limiting oxygen index (LOI) of only 17.9%; thus, it is flammable and releases toxic gases during combustion, severely restricting its application in high-security scenarios [4,5,6]. Recent studies have focused on synergistic flame-retardant strategies to balance the flame-retardant performance and material properties [7,8]. Early physical blending methods inhibited combustion via the addition of inorganic (e.g., aluminum hydroxide) or intumescent flame retardants (e.g., ammonium polyphosphate); however, the high additive quantities generally deteriorated the mechanical properties. Chemically reactive flame retardants enhance the intrinsic flame retardancy by bonding to phosphorus, nitrogen, and other elements, but they face synthetic complexity and cost limitations [9,10]. The current trend is generally a physicochemical synergistic system combined with nano-enhancement technology to optimize the efficiency and functionality of flame retardancy [11,12,13].

A flame retardant is categorized as physically added or chemically reactive, according to its mechanism of action. The former inhibits combustion via decomposition based on heat absorption (e.g., the dehydration of Al(OH)_3_), gas-phase dilution, or the provision of a cohesive-phase charcoal barrier [14,15]. This is a simple process, but it requires a high amount of additive and generally embrittles the material. The latter releases a free-radical quencher (e.g., PO–) or catalyzes charcoal formation via chemical bonding, yielding two-phase flame retardancy, but its synthesis is complicated [16,17]. The composite application of two types of flame retardants (e.g., synergistically applying an intumescent flame retardant and nanoclay) can reduce the amount of additive and enhance the densification of the carbon layer [18,19,20]. This is an effective method for use in the flame-retardant modification of PEO.

Calcium sulfate whisker (CSW), which is a novel type of flame-retardant filler, has attracted considerable attention because of its high aspect ratio, thermal stability, and green properties [21,22,23]. Its flame-retardant mechanism originates from the release of crystalline water at high temperatures to absorb heat and provide a cooling effect, and the combination of residual CaSO_4_ particles with the carbon layer to form a thermal barrier [24,25]. However, the enhancement of the flame retardancy of PEO combined with CSW is limited because of its lack of a gas-phase radical-trapping capacity and its weak interfacial bonding [26,27]. In this study, we combine calcium stearate-modified CSW (MCSW) with the phosphorus-based flame retardant 1,4-bis(diphenoxyphosphoryl)piperazine (DIDOPO) [28] to yield a synergistic gas phase-condensed phase system. MCSW is a modified CSW that can enhance the interfacial bonding capacities of PEOs [29]. MCSW prevents agglomeration by enhancing the interfacial compatibility, and DIDOPO interrupts the chain reaction by releasing PO– radicals at the early stage of combustion and promotes the generation of aromatic carbon. Adding 10% MCSW and 5% DIDOPO increased the LOI of the PEO composite from 17.9% to 26.5%, and the UL-94 rating reached V-0. Additionally, the respective peak heat release rate (p-HRR) and total heat release (THR) were reduced by 38.9% and 20.07%, and the mechanical properties were not significantly degraded.

This study provides an efficient synergistic strategy for use in the flame-retardant modification of PEO and extends it to other flammable polymer systems. In the future, optimizing the surface functionalization of MCSW, exploring the compounding effects of bio-based flame retardants [30,31,32], and enhancing their durability by combining them with dynamic cross-linking technology shall be necessary. Addressing these points shall promote the practical application of PEO-based materials in batteries and other industrial fields.

## 2. Materials and Methods

### 2.1. Materials

PEO (Tianjin Komeo Chemical Reagent, Tianjin, China), hydrochloric acid (AR analytically pure, Sinopharm Chemical Reagent, Shanghai, China), anhydrous ethanol (AR analytically pure, Sinopharm Chemical Reagent), calcium stearate (Shanghai Macklin Biochemical, Shanghai, China), magnesium nitrate hexahydrate (Tianjin Komeo Chemical Reagent), phosphogypsum (Guizhou Kai Phosphorus, Guiyang, China), and DIDOPO (prepared in the laboratory) [28] were obtained.

### 2.2. Preparation of Phosphogypsum-Extracted CSWs

**Figure 1:**
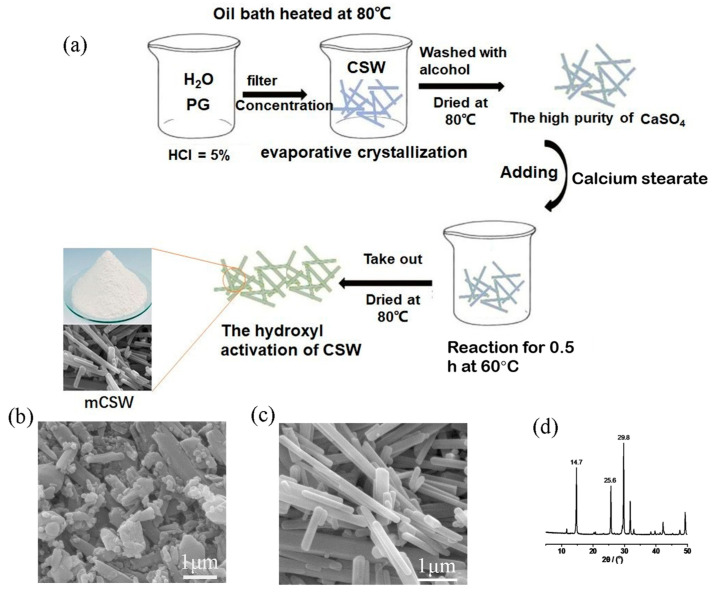
(**a**) Flowchart of the preparation of MCSW, (**b**) SEM image of phosphogypsum (PG), and (**c**,**d**) SEM image and X-ray diffraction pattern of CSW.

Phosphogypsum (Figure 1b) was placed in a drying box in advance, and a 5% hydrochloric acid solution was prepared and stored (hydrochloric acid content of 36–38%, 69.4 mL, 430.6 mL of purified water). Phosphogypsum (15 g) was ground with 300 mL of 5% hydrochloric acid and then poured into a 500 mL flask, which was placed onto a collector-type hot-plate magnetic stirrer to react for 40 min (over this time, 3 × ≤0.2 g of magnesium nitrate was added to increase the temperature to 80 °C). The resulting solution was filtered using a pumping machine, and the filtrate was collected and poured into a 500 mL beaker with a 99-1A high-power magnetic stirrer with a digital thermostat for concentration (30 min at 80 °C). The solution was then filtered (and washed with anhydrous ethanol) to yield the filter cake, which was oven-dried, ground, and stored in a sealed bag (Figure 1a). As shown in Figure 1d, three strong diffraction peaks are observed at 2θ = 14.7°, 25.6°, and 29.8°, representing the (100), (110), and (111) crystal planes of the CSW, respectively. In addition, the CSWs are needle-like, with diameters of approximately 3–12 μm, smooth surfaces, and no defects. A flowchart of the preparation of MCSW is shown in Figure 1c.

### 2.3. Preparation of MCSW

CSW (1.96 g) and 0.04 g of calcium stearate were weighed using a balance and then mixed with 100 mL of anhydrous ethanol (calcium stearate-modified, 2%) in a beaker. The mixture was transferred to a round-bottomed flask and reacted for 30 min at 60 °C, and then the liquid was filtered via low-temperature vacuum extraction. The obtained cake was dried at 60 °C in an electric blast oven for 24 h, and then the powder was ground and packed into a sealed bag for further use.

### 2.4. Preparation of PEO/DIDOPO/MCSW

PEO, DIDOPO, and MCSW were dried at 85 °C for 5 h, and then the PEO/DIDOPO/MCSW composites were melt-mixed using a torque rheometer at 150 °C (Haake PolyLab OS, Thermo Fisher Scientific, Waltham, MA, USA). The compositions of the samples are shown in Table 1. The compounds were initially hot-pressed (16 MPa) at 150 °C for 12 min. They were then utilized to prepare sheets of appropriate sizes and thicknesses using a plate vulcanizing machine (ZHY-W-1, Chengde Testing Machine Factory, Chengde, China) at 25 °C and 16 MPa for 20 min.

### 2.5. Characterization

Fourier transform infrared (FTIR) spectroscopy (Nicolet iS50, Thermo Fisher Scientific) of CSW and MCSW was performed in a wavenumber range of 400–4000 cm^–1^.

The UL-94 vertical combustion study was performed according to ASTM D3801 [33] with a sample size of 130.0 × 13.0 × 3.2 mm. The burning times after the first (t_1_) and second ignition (t_2_) of each formulation are the means of five measurements.

The LOIs were evaluated using an LOI instrument according to ASTM D2863-06. The dimensions of the sample were 130.0 × 6.5 × 3.2 mm. The cone calorimeter studies were conducted using a Fire Testing Technology (East Grinstead, UK) cone calorimeter under an external heat flux of 50 kW/m^2^ according to ISO 5660-1 (specimen size of 100.0 × 100.0 × 6.0 mm) [34]. Each sample was evaluated thrice, and the typical results were reproducible within ±10%.

The thermal properties of the samples were analyzed via thermogravimetric analysis (TGA, TG 209 F3 Tarsus, Netzsch, Selb, Germany) in a nitrogen atmosphere at a flow rate of 60 mL/min. Each sample (approximately 6–10 mg) was heated from room temperature to 700 °C at 10 °C/min to yield the TGA and differential thermogravimetry (DTG) thermograms.

The surface morphology of the char residue after the cone calorimeter study was determined using scanning electron microscopy (SEM, Quanta FEG 250, FEI, Hillsboro, OR, USA) at an acceleration voltage of 20 kV.

The rheological properties of the composites were determined using a rheometer (HAAKE MARS II, Thermo Fisher Scientific) with a parallel-plate geometry (respective diameter and thickness of 25 and 1 mm). Frequency scans were performed from 0.01 to 100 rad/s at a strain of 1%, with measurement at 130 °C.

The samples were analyzed using energy-dispersive X-ray spectroscopy (EDS, INCA Energy 350, Oxford Instruments, Abingdon, UK) at 20 kV.

The gases released during TGA were investigated using the Nicolet iS50 FTIR spectrometer. TGA-FTIR analysis was conducted using TGA coupled with FTIR spectroscopy. The FTIR spectrum of each sample was collected over 16 scans in the wavenumber range 500–4000 cm^−1^.

## 3. Results and Discussion

### 3.1. Characterization of MCSW

**Figure 2:**
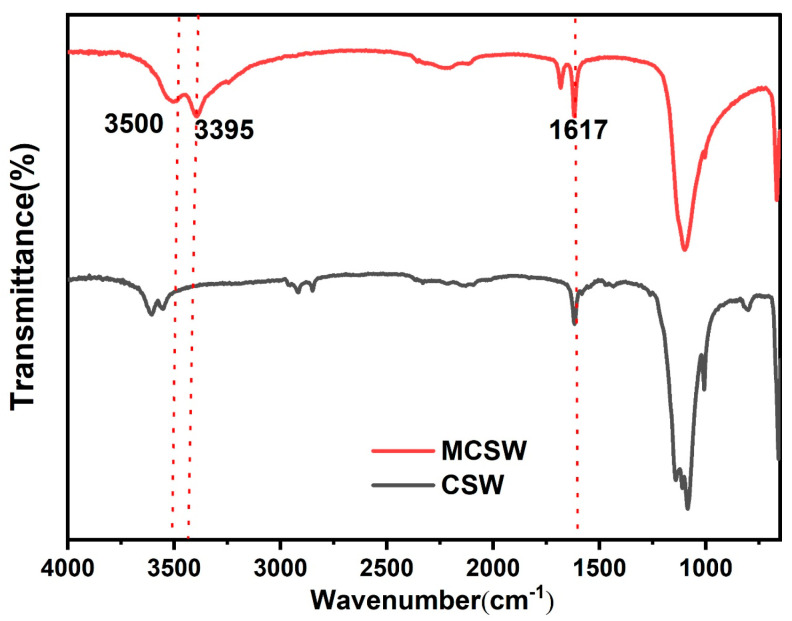
FTIR spectra of CSW and MCSW.

Figure 2 shows the FTIR spectra of CSW and MCSW. As shown in Figure 2, the peak in the spectrum of CSW at 1617 cm^–1^ represents the symmetric bending vibration of O–H. Peaks representing the O–H of MCSW at 3500 and 3395 cm^–1^ are clearly observed, whereas the spectrum of the original CSW displays no such signals in this region. These signals indicate that the modification of CSW is successful, excluding the possibility of pure physical adsorption and directly reflecting the chemical bonding of calcium stearate to the active sites on the surface of CSW. Calcium stearate successfully modifies the surface of CSW, providing hydrophobicity and functionalization.

**Figure 3:**
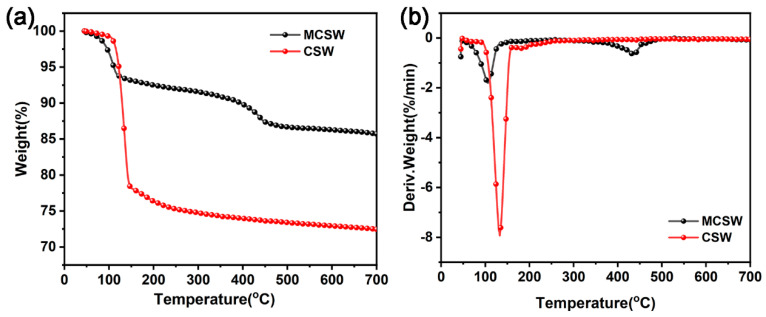
TGA (**a**) and DTG (**b**) thermograms of CSW and MCSW under an N_2_ atmosphere.

Comparing the results of TGA, as shown in Figure 3 and Table 2, yields the following conclusion: the initial thermal stability of MCSW is slightly decreased, and its initial decomposition temperature (T_5%_, 111.3 °C) is approximately 10 °C lower than that of CSW (121.8 °C). This may be attributed to the low-temperature decomposition of the alkyl chains or residual organic components of calcium stearate, which indirectly confirms the presence of the modifier. However, the main decomposition temperature T_max-1_ of MCSW (133.3 °C) is significantly higher than that of CSW (106.8 °C); thus, the thermal stability of the modified material is enhanced in the low-temperature region. This may be attributed to the formation of a stable interfacial layer between calcium stearate and the surface of CSW via chemical bonding, which suppresses the early pyrolysis of the matrix. Notably, the pyrolysis peak (T_max-2_) of the original CSW at 437.6 °C is no longer observed for MCSW, suggesting that the pyrolysis pathway of the modified material is reconfigured. Calcium stearate may hinder further decomposition at high temperatures by covering the active sites or changing the crystalline structure of the inorganic phase. In addition, the amount of residual char of MCSW (85.71 wt.%) is significantly higher than that of CSW (72.44 wt.%) at 700 °C. This suggests that introducing calcium stearate not only enhances the interfacial bonding but also significantly improves the high-temperature resistance of the material to thermal decomposition by promoting charring or inhibiting the release of volatile products. Based on these changes in thermal behavior, the results suggest that the surface of CSW is chemically modified with calcium stearate, endowing it with superior thermal stability and carbonization properties.

### 3.2. Flame Retardancies of the PEOs

Upon analyzing the flame retardancy data shown in Table 1, four samples, i.e., PEO, PEO/15% DIDOPO, PEO/15% MCSW, and PEO/10% DIDOPO/5% MCSW, were used in subsequent experiments, based on the following.

Pure PEO exhibits an LOI of only 17.9% and no UL-94 rating [35]; thus, it fails the vertical combustion test and is highly flammable and indispensable as a benchmark. Upon adding 15% DIDOPO, the LOI increases to 23.8% and the UL-94 reaches a rating of V-0, confirming that DIDOPO, as a vapor-phase flame retardant, effectively inhibits the combustion chain reaction via the release of phosphorus radicals. Hence, DIDOPO is a typical single flame retardant. When 15% MCSW is added alone, the LOI increases to 25.3% (exceeding the 23.8% of DIDOPO addition alone), and the UL-94 also reaches a rating of V-0. Therefore, MCSW, as a condensed-phase flame retardant, significantly improves the flame-retardant efficiency via the formation of a CaSO_4_ insulation layer and decomposition based on heat absorption; its effect alone is superior and must be included in the comparison. The LOI of PEO/10% DIDOPO/5% MCSW reaches 26.5% (the highest LOI among those of the compounded samples), and a UL-94 grade of V-0 is maintained. Thus, the gas-phase free radical quenching of DIDOPO and condensed-phase heat insulation of MCSW produce a synergistic effect at this ratio. Although the synergistic effect is weak, it not only reduces the total amount of flame retardant additives, but also realizes a breakthrough in performance. In contrast, although the other compounding ratios are improved, the flame-retardant grades and LOIs do not exceed those of the above four samples. Therefore, the optimal synergistic sample, i.e., PEO/10% DIDOPO/5% MCSW, was used to determine the synergistic mechanism. In conclusion, the four samples cover the unmodified benchmark, optimal performance of a single flame retardant, and optimal synergistic combination. Hence, the differences in the effects of the gas, condensed, and synergistic flame-retardant strategies can be systematically compared and a clear direction can be provided for the subsequent study of flame-retardant mechanisms and material optimization.

### 3.3. Cone Calorimetry

Flame inhibition effect = 1 − EHCFR-MCSW/EHC MCSW,(1)

Barrier protective effect = 1 − (p-HRRFR-MCSW/p-HRR MCSW)/(THRFR-MCSW/THREP),(2)

Charring effect = 1 − TMLFR-MCSW/TML MCSW,(3)

**Figure 4:**
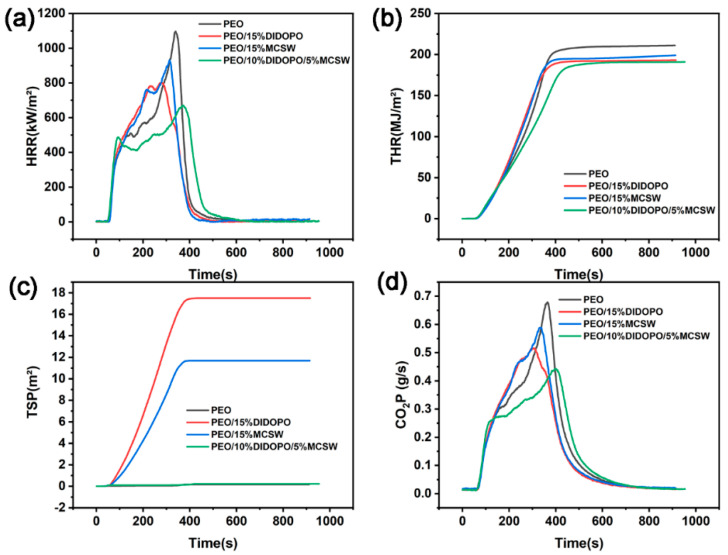
HRR (**a**), THR (**b**), TSP (**c**), and carbon dioxide production (**d**) curves of the PEOs.

The mechanism whereby MCSW and DIDOPO synergistically enhance the flame retardancy of PEO is revealed by the results of cone calorimetry, as shown in Figure 4 and Table 3. Pure PEO displays a respective time to ignition (TTI), p-HRR, and THR of 47 s, 1097.8 kW/m^2^, and 211.01 MJ/m^2^; thus, it is highly flammable and burns vigorously. Upon adding 15% DIDOPO, the TTI is extended to 53 s, the p-HRR is significantly reduced to 801.28 kW/m^2^, and the THR is reduced to 193.21 MJ/m^2^. This confirms the effectiveness of DIDOPO in quenching the combustion chain reaction in the gas phase via the release of phosphorus radicals (PO–). However, the total smoke production (TSR) surges from 0.174 m^2^/m^2^ for pure PEO to 17.513 m^2^/m^2^, and the CO_2_ production rate (CO_2_P) is 5.336 g/s. This suggests that the single vapor-phase flame retardant leads to incomplete combustion, exacerbating the release of smoke and toxins. In contrast, the TTI of the system with 15% MCSW alone is also extended to 53 s, the p-HRR decreases to 931.31 kW/m^2^, and the THR is 199.07 MJ/m^2^. Therefore, the MCSW absorbs heat via high-temperature decomposition to generate CaSO_4_ particles and water of crystallization and forms a physical insulation layer in the condensed phase. However, the TSR (11.687 m^2^/m^2^) remains high and the CO_2_P (6.0042 g/s) is close to that of pure PEO; thus, a single condensed-phase barrier displays limited smoke and toxicity control.

When DIDOPO (10%) is compounded with MCSW (5%), the synergistic effect is fully highlighted: the p-HRR is further reduced to 670.58 kW/m^2^ and the THR is 190.94 MJ/m^2^, which is significantly superior to those observed upon adding a single component. Hence, MCSW compensates for the structural defects of DIDOPO by filling the pores of the carbon layer and forming a dense CaSO_4_ barrier. Conversely, DIDOPO reduces the generation of combustible gases via free radical trapping and both synergistically inhibit heat transfer and energy release. In terms of smoke control, the TSR of the compound system is only 0.22 m^2^/m^2^, which is similar to that of pure PEO (0.174 m^2^/m^2^) and considerably lower than that of the system with DIDOPO alone (17.513 m^2^/m^2^). Thus, the physical barrier effect of MCSW effectively inhibits the smoke release caused by DIDOPO. The CO_2_P of the compound system is significantly lower than that of pure PEO; thus, MCSW can reduce the amount of combustibles, and the compound system is a good option for controlling the smoke. Simultaneously, the significantly lower CO_2_P compared to that of pure PEO confirms that MCSW reduces the release of toxic gases by reducing the decomposition of the combustible materials. Although the average effective heat of combustion (av-EHC) of the composite system is slightly elevated, it realizes a more complete combustion pathway via synergistic carbon formation, and the significant optimization of the heat release parameters (p-HRR and THR) still ensures the overall flame-retardant efficiency.

In summary, the synergistic effect of MCSW and DIDOPO is reflected in the complementary “gas phase–condensed phase” dual-phase flame-retardant mechanism: DIDOPO dominates the gas-phase free radical quenching and inhibits flame propagation. MCSW fills the cavities of the carbon layer with the CaSO_4_ insulation layer to block heat and oxygen transfer and mitigate the smoke emission of DIDOPO, and alleviates the smoke and toxic side effects of DIDOPO. The optimization of the p-HRR, THR, TSR, and other key indices of the composite system confirms the viability of MCSW as a synergist to enhance the flame retardancy of PEO. This provides a critical experimental basis for the development of highly efficient and low-smoke and -toxicity flame-retardant materials [36,37,38].

Table 4 confirms the mechanism whereby MCSW synergistically enhances the flame retardancy of PEO with DIDOPO [39]. After adding 15% DIDOPO to the pure PEO matrix, the flame suppression effect (10.7%) indicates that it is effective in quenching the combustion chain reaction by releasing phosphorus radicals (PO–) in the gas phase. However, the barrier effect (20.3%) and rate of char formation (1.1%) are low, suggesting that forming a stabilized char layer with DIDOPO only is challenging. Conversely, the flame suppression effect of adding 15% MCSW alone is only 3.5%, but the rate of char formation increases to 3.2%. Hence, MCSW generates CaSO_4_ particles via high-temperature decomposition and initially forms a thermal barrier in the condensed phase; however, the barrier effect (10.1%) remains limited owing to the lack of gas-phase flame-retardant components. When DIDOPO (10%) is compounded with MCSW (5%), the flame suppression effect decreases slightly to −0.01%, likely due to the physical filling of MCSW interfering slightly with the gas-phase radical trapping of DIDOPO. However, the barrier effect (32.5%) and char formation rate (9.8%) are significantly improved. DIDOPO catalyzes cross-linking to generate the char precursor. MCSW produces char via CaSO_4_ particles to fill the pores of the carbon layer, and the high-aspect-ratio MCSW fiber enhances the mechanical strength of the carbon layer. These phenomena synergistically form a dense, continuous multilayer barrier to effectively isolate the heat and oxygen. In addition, the heat-absorbing decomposition (release of crystalline water) of MCSW further lowers the system temperature, which compensates for the insufficiency of DIDOPO in condensed-phase flame retardation. Although the gas-phase inhibition efficiency of the compounded system fluctuates slightly, its barrier effect is 60% higher than that of the system with DIDOPO only. Additionally, the rate of char formation is 206% higher than that of the system with MCSW only; thus, the synergistic mechanism of physical enhancement and thermal modulation using MCSW successfully compensates for the limitations of a single flame retardant.

### 3.4. Thermal Stability Analysis

**Figure 5:**
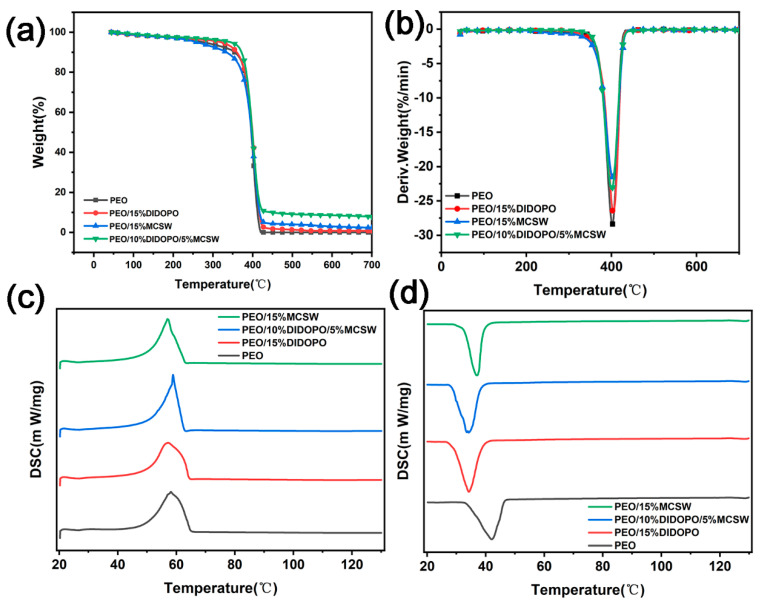
(**a**,**b**) TGA curves of PEOs, and (**c**,**d**) the melting and crystallization curves of PEOs in a N2 atmosphere.

The results of TGA, as shown in Figure 5 and Table 5, reveal the mechanism whereby MCSW and DIDOPO synergistically enhance the thermal stability of PEO and its capacity to form char. The T_5%_ of pure PEO is 273.96 °C, and the temperature corresponding to the maximum decomposition rate (T_max_) is 401.5 °C. Decomposition is almost complete at 700 °C; thus, it exhibits poor thermal stability and lacks a flame-retardant carbon layer. Adding 15% DIDOPO increases the T_5%_ to 321.07 °C; thus, it retards the initial decomposition by releasing phosphorus radicals, but the amount of residual charcoal remains 0 wt.%, suggesting that its gas-phase flame-retardant mechanism cannot promote charcoal formation. Conversely, adding 15% MCSW alone marginally increases the T_5%_ to 295.45 °C, and the amount of residual charcoal is increased to 2.31%. Hence, MCSW decomposes at high temperatures to generate CaSO_4_ particles, initially forming a heat-insulating carbon layer, but the enhancement of the thermal stability is limited. When DIDOPO (10%) is compounded with MCSW (5%), the synergistic effect is significant: the T_5%_ increases to 344.51 °C, and the amount of residual carbon increases drastically to 7.82%. Thus, DIDOPO retards decomposition and catalyzes the cross-linking of the carbon in the gas phase, whereas MCSW forms a dense, continuous physical barrier by filling the pores of the carbon layer and stabilizing the structure. In addition, the T_max_ of the complex system (403.5 °C) is similar to that of pure PEO (401.5 °C); thus, the main decomposition phase is not significantly altered. However, an increased mass is retained as residual char at high temperatures rather than during complete decomposition into combustible gas. Therefore, the “gas phase–condensed phase” synergistic mechanism of MCSW and DIDOPO significantly improves the thermal stability of PEO.

Figure 5 and Table 5 show the results of the TGA and DSC of the PEOs, with different filler systems showing the differential modulation of the crystallization and melting behaviors of the PEO-based composites. The melting temperature (T_m_) of pure PEO is 58.15 °C, and the cold crystallization temperature (T_c_) is 42.07 °C. When 15% DIDOPO is introduced, the T_m_ decreases slightly to 57.22 °C, whereas the T_c_ decreases significantly to 34.26 °C. Therefore, the rigid molecular structure of DIDOPO may lower the energy barrier of cold crystallization by enhancing the motilities of the chain segments. In addition, its heterogeneous nucleation effect weakens the perfection of the PEO crystal. In contrast, introducing 15% MCSW reduces the T_m_ and T_c_ to 57.00 and 37.03 °C, respectively. Although its lamellar structure slightly inhibits the lattice regularity, it accelerates cold crystallization more weakly than DIDOPO, which may be related to the interfacial hydrogen-bonding effect restricting the rearrangement of the molecular chains. Notably, when 10% DIDOPO and 5% MCSW are introduced, the T_m_ paradoxically increases to 58.88 °C (0.73 °C higher than that of pure PEO), and the T_c_ decreases further to 34.17 °C. The synergistic effect between the fillers may induce a more perfect crystal structure (e.g., the optimization of the heterogeneous nucleation density), and the respective plasticizing and confined-space effects of DIDOPO and MCSW reduce the kinetic resistance of crystallization.

### 3.5. TGA-FTIR Analysis

**Figure 6:**
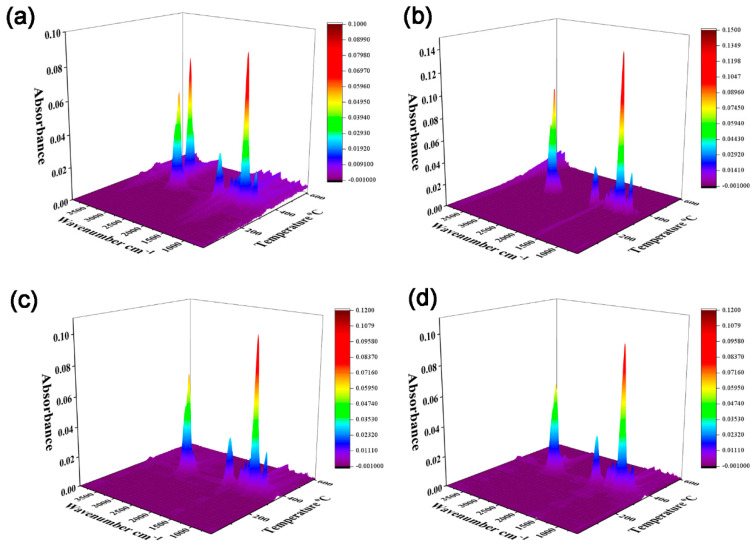
Three-dimensional TGA-FTIR spectra of (**a**) PEO, (**b**) PEO/15% DIDOPO, (**c**) PEO/10% DIDOPO/5% MCSW, and (**d**) PEO/15% MCSW.

The TGA-FTIR spectra of the PEO, PEO/15% DIDOPO, PEO/15% MCSW, and PEO/10% DIDOPO/5% MCSW composites at different decomposition temperatures are shown in Figure 6 to explore the changes in the pyrolysis gas products in detail. At 300–400 °C, peaks are observed at 2864 and 2924 cm^–1^, and the peaks observed at 1746, 1130 and 1230, 1085, and 881 cm^–1^, respectively, represent carbonyl compounds, aliphatic esters, and C–O and C–H bonds. In addition, the peak at 3500–4000 cm^–1^ is attributed to water. When 10% DIDOPO/5% MCSW is added to PEO, absorption peaks are observed at 1230 cm^–1^, which are attributed to P–O, along with the characteristic peaks of C–O (1085 cm^–1^) and C=O (1130 cm^–1^). This suggests that introducing MCSW only is ineffective in increasing the gas-phase products. The composite addition of DIDOPO and MCSW results in the manifestation of the absorption peaks of phosphorus-containing groups and increases in the amounts of gas-phase volatiles during the thermal degradation of the blend.

**Figure 7:**
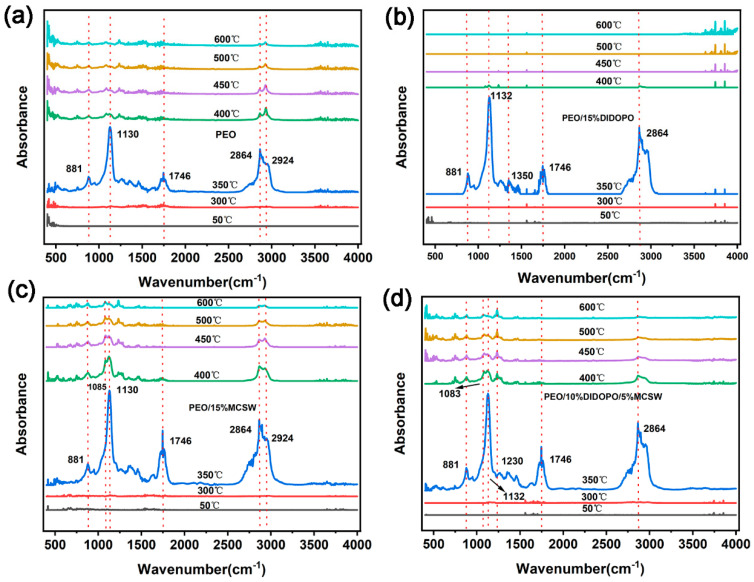
TGA-FTIR spectra of the gaseous phases during the thermal decomposition of (**a**) PEO, (**b**) PEO/15% DIDOPO, (**c**) PEO/15% MCSW, and (**d**) PEO/10% DIDOPO/5% MCSW.

The dynamic evolution of the chemical structures and thermal stabilities of PEO and its composite system during pyrolysis were systematically analyzed via FTIR coupling. As shown in Figure 7, the spectrum of pure PEO displays typical C–H bending (881 cm^–1^), C–O–C backbone (1130 cm^–1^), carbonyl (1746 cm^–1^), and C–H stretching (2864 cm^–1^) vibrations, which gradually decay and disappear as the temperature increases to 400 °C. This confirms the complete decomposition of the PEO molecular chains at high temperatures. In the spectrum of the PEO/15% DIDOPO system, the characteristic P=O (1230 cm^–1^) and P–O–C (1132 cm^–1^) peaks of DIDOPO are still observable at 350 °C. However, their rapid disappearance at >400 °C reveals the limitation of a single gas-phase flame retardant during high-temperature protection. Notably, the characteristic C–O (1085 cm^–1^) and C=O (1130 cm^–1^) peaks of MCSW can still be detected at 500 °C in the spectrum of the PEO/15% MCSW system; thus, the condensed-phase catalytic effect of MCSW can significantly retard the decomposition of the polymers. The synergistic system PEO/10% DIDOPO/5% MCSW exhibits a unique dual-protection mechanism: the characteristic P–O–C (1230 cm^–1^) and C–O (1130 cm^–1^) peaks of DIDOPO and MCSW, respectively, maintain high signal intensities at 350 °C and remain detectable at 600 °C. Therefore, MCSW can significantly retard the decomposition of the polymers via condensed-phase catalysis. The respective high signal intensity and detectability at 350 and 600 °C are attributed to the release of phosphorus-containing radicals from DIDOPO and MCSW synergistically forming a dense carbon layer with an enhanced thermal stability. This synergistic system provides multilevel protection against polymer pyrolysis via the coupling of gas-phase flame retardation (radical quenching) and a condensed-phase barrier (char-layer protection), providing direct evidence of the synergistic mechanism of flame retardation at the molecular level.

### 3.6. Analysis of the Char Residues

**Figure 8:**
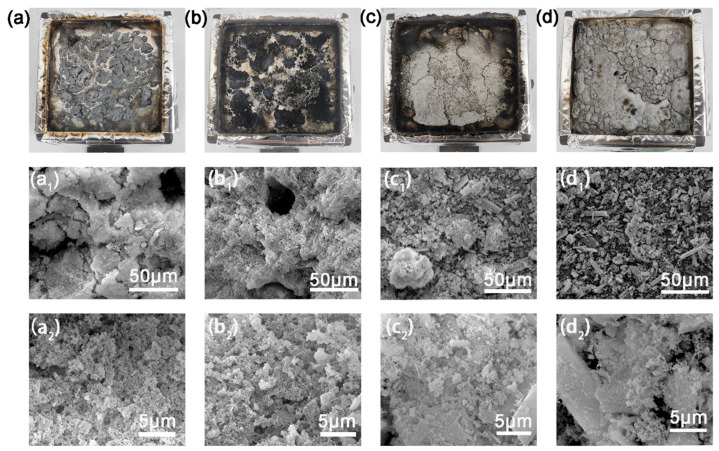
Digital images of the char residues of (**a**,**a_1_**,**a_2_**) PEO, (**b**,**b_1_**,**b_2_**) PEO/15% DIDOPO, (**c**,**c_1_**,**c_2_**) PEO/10% DIDOPO/5% MCSW, and (**d**,**d_1_**,**d_2_**) PEO/15% MCSW after cone calorimetry.

As shown in Figure 8, the MCSW compensates for the defects in the carbon layer caused by the DIDOPO vapor-phase flame retardant via the coalescence phase, which significantly improves the flame-retardant performance. DIDOPO, as a phosphorus flame retardant, mainly releases PO– radicals in the vapor phase, quenches the reactive radicals of the combustion chain reaction (e.g., H– and OH–), and effectively inhibits flame propagation. However, its effect is limited to the vapor phase, and forming a dense carbon layer in the coalescence phase is challenging, which leads to the formation of a carbon layer with pores or a loose structure. These voids weaken the thermal insulation performance of the carbon layer, such that heat and oxygen can still penetrate it, limiting the efficiency of flame retardancy. Conversely, MCSW decomposition at high temperatures generates CaSO_4_ particles and releases crystalline water, which optimizes the structure of the carbon layer via a dual mechanism. The CaSO_4_ particles fill the pores that are not covered by DIDOPO (SEM images shown in Figure 8a–c) [40], forming a continuous, dense physical barrier and blocking heat and mass transfer. Meanwhile, the high aspect ratio of the fiber structure of MCSW results in a loose structure, strengthening the thermal insulation performance of the carbon layer. The high-aspect-ratio fiber structure enhances the mechanical strength of the carbon layer, preventing crack expansion owing to thermal stress during combustion; e.g., the SEM images of the PEO/10% DIDOPO/5% MCSW system indicate that the surface of the carbon layer is smooth and its porosity is low (Figure 8(c_1_,c_2_)); thus, MCSW effectively compensates for the structural defects caused by the single gas-phase action of DIDOPO. In addition, the decomposition (release of the crystalline water) of MCSW based on heat absorption further reduces the system temperature, which synergizes with the gas-phase radical capture of DIDOPO to yield the dual flame retardancy of the gas phase–condensed phase.

### 3.7. Elemental Analysis of the Char Residues

**Figure 9:**
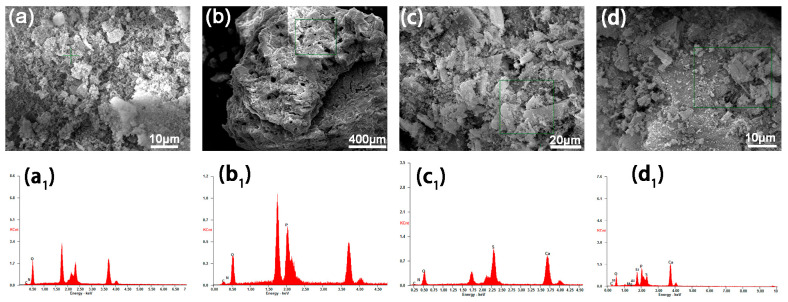
(**a**–**d**,**a_1_**–**d_1_**) Respective EDS plots of PEO, PEO/15%DIDOPO, PEO/15%MCSW, and PEO/10%DIDOPO/5%MCSW.

Based on the elemental compositions shown in Table 6 and Figure 9, the flame retardant synergistic mechanism of the PEO/10% DIDOPO/5% MCSW composite system can be explained in detail by the characteristics of the elemental distribution: the contents of carbon (10.4 wt.%) and oxygen (85.18 wt.%) and the small amount of nitrogen (4.42 wt.%) within pure PEO reflect its linear polymeric nature [41]. Adding 15% DIDOPO significantly increases the respective phosphorus and carbon contents to 28.93 and 13.57 wt.%; in contrast, the introduction of 15% MCSW provides the system with a typical calcium sulfate profile (S: 21.24 wt.%, Ca: 29.08 wt.%), and catalytic dehydrogenation sharply reduces the carbon content to 3.94 wt.%. However, no P is detected, suggesting that MCSW relies on the insulation layer generated via the decomposition of the sulfate with the quenching of the free radicals [42]. Notably, the phosphorus content (13.68 wt.%) of the synergistic system is significantly lower than that of the pure DIDOPO system. The sulfur (8.82 wt.%) and calcium (26.97 wt.%) contents are also lower than those of the pure MCSW system, with an intermediate carbon content (6.54 wt.%) and a significant decrease in the oxygen content (33 wt.%) using the combined system. This reveals that the chemistries of DIDOPO and MCSW are inferior to that of the synergistic system. This partially immobilizes the phosphorus (leading to a decrease in the detectable free phosphorus) and increases the amount of the residual phase of the calcium sulfate (S/Ca ≈ 0.33, which is similar to the non-stoichiometric ratio of the decomposition product of CaSO_4_) within the “organic–inorganic hybrid carbon layer”. This multiscale structure reduces the amount of carbon residue by 37% compared to that of pure PEO. Simultaneously, it multiplies the efficacies of free-radical trapping (gas phase) and the inhibition of ceramization (cohesive phase) via the ternary synergy of phosphorus–sulfur–calcium. This forms a logically closed loop with the enhanced amount of residual carbon and optimized thermal stability of this system, as observed in the preliminary thermal analysis. This also provides key experimental evidence of the chemical nature of the flame-retardant synergy.

### 3.8. Mechanical Properties of the PEO Composites

**Figure 10:**
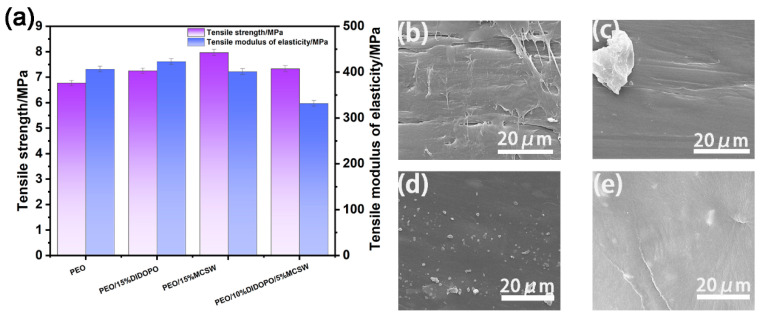
(**a**) Histogram of the mechanical properties of the PEOs. (**b**–**e**) Cross sections of PEO, PEO/15% DIDOPO, PEO/15% MCSW, and PEO/10% DIDOPO/5% MCSW, respectively.

As shown in Figure 10, the tensile strengths and elastic moduli of the PEO materials change significantly upon introducing the additives. Compared to those of pure PEO, adding 15% DIDOPO increases the tensile strength and elastic modulus from 6.77 to 7.25 MPa and 406.60 to 423.02 MPa; thus, adding DIDOPO enhances the mechanical properties of the material. However, when 10% DIDOPO and 5% MCSW are added simultaneously, the tensile elastic modulus decreases drastically to 331.79 MPa, although the tensile strength increases further to 7.33 MPa. This may be attributed to the alteration of the microstructure of the material via the incorporation of MCSW, resulting in a decrease in its rigidity. In contrast, the sample with 15% MCSW alone displays the highest tensile strength of 7.97 MPa and a tensile elastic modulus of 401.22 MPa, which is slightly lower than that of pure PEO, but the overall mechanical properties are superior. The effects of the different additives on the surface and fracture surface of the material can be further observed in the micromorphology images shown in Figure 10b–e. The surface morphologies and fracture characteristics of the samples differ significantly, and these microstructural changes correspond to the changes in the mechanical properties, providing a crucial basis for understanding the mechanisms of action of the additives within the PEO matrix.

### 3.9. Analysis of the Rheological Properties

**Figure 11:**
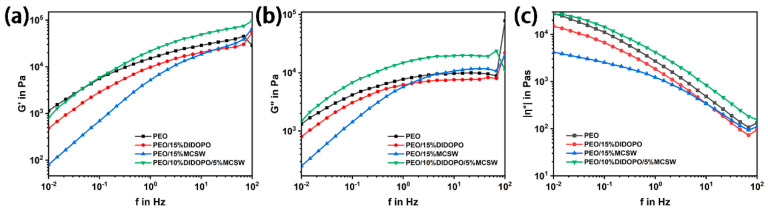
Storage (**a**), loss moduli (**b**) and complex viscosities (**c**) of the PEOs.

The multiscale structural evolution of the PEO-based composites was revealed via rheological analysis (Figure 11) [43,44]. Compared to those of the pure PEO system, the composites with 15% DIDOPO or 15% MCSW display significant decreases in their energy storage (G′) and loss moduli (G″) and complex viscosities (|η|). These decreases are attributed to the plasticizing effect of DIDOPO weakening the molecular chain entanglement, while the weak interfacial binding of the MCSW nanoparticles leads to the loosening of the network structure. However, the PEO/10% DIDOPO/5% MCSW synergistic system exhibits paradoxically enhanced rheological properties, with increases in G′, G″, and |η| compared to those of PEO. This may be attributed to the cross-scale synergy between DIDOPO and MCSW—the phosphate group of DIDOPO combines with a hydroxyl group on the calcium sulfate surface of MCSW via hydrogen bonding to form an “organic–inorganic hybrid physical cross-linking point”, which significantly enhances the intermolecular interactions. The inversions of the rheological properties confirm the P-Ca-S synergistic mechanism of carbon formation elucidated via the elemental and thermal analyses. Thus, the chemical compatibility and topological matching between the flame retardant and filler are key in regulating the performance of polymer processing and service performance.

## 4. Conclusions

In this study, a flame-retardant PEO composite was prepared via the melt doping of PEO with a mixture of DIDOPO and MCSW. The effects of MCSW on the thermal stability of the sample, in addition to the morphology and compositional structure of the residual carbon after combustion, were systematically investigated using different analytical techniques. PEO containing 10 wt.% DIDOPO and 5 wt.% MCSW exhibited a UL-94 rating of V-0. Cone calorimetry indicated that adding MCSW significantly reduced the p-HRR and THR and increased the amount of residual carbon, resulting in superior flame-retardant and smoke-suppression properties compared to those of PEO containing 15 wt.% DIDOPO alone. Increases in the T_5%_ and residue content were observed via TGA after adding MCSW mixed with DIDOPO to PEO. The morphologies of the residues indicated that introducing MCSW facilitated the formation of a tighter, more stable, and coherent carbon layer, which effectively inhibited smoke generation and heat release during combustion. Additionally, the tensile strength of the PEO/DIDOPO/MCSW composite was superior to that of PEO. In conclusion, MCSW can be used as a promising, efficient synergist in combination with DIDOPO. The flame-retardant and smoke-suppression properties of the flame-retardant PEO were significantly improved, and its mechanical properties were maintained.

## Figures and Tables

**Table 1:** Results of the UL-94 and LOI studies of the PEOs.

Sample	PEO (wt.%)	DIDOPO (wt.%)	MCSW (wt.%)	Flame Retardancy	
				LOI (%)	UL-94
PEO	100	0	0	17.9	No Rating
PEO/15% DIDOPO	85	15	0	23.8	V-0
PEO/14% DIDOPO/1% MCSW	85	14	1	24.5	V-0
PEO/12% DIDOPO/3% MCSW	85	12	3	25.0	V-0
PEO/10% DIDOPO/5% MCSW	85	10	5	26.5	V-0
PEO/8% DIDOPO/7% MCSW	85	8	7	24.8	V-1
PEO/15% MCSW	85	0	15	25.3	V-0

**Table 2:** Results of the TGA and DTG of CSW and MCSW.

Sample	T_5%_	T_max-1_	T_max-2_	Char Residue at 700 °C
	(°C)	(°C)	(°C)	(wt.%)
CSW	121.8	106.8	437.6	72.44
MCSW	111.3	133.3	-	85.71

**Table 3:** Results of the cone calorimetry of the PEOs.

Sample	TTI	Average Heat Release Rate	p-HRR	THR	TSR	av-EHC	CO_2_P	TML
	(s)	(kW/m^2^)	(kW/m^2^)	(MJ/m^2^)	(m^2^/m^2^)	(MJ/kg)	(g/s)	(wt.%)
PEO	47	507.38	1097.8	211.01	0.174	30.446	6.02	99.4
PEO/15% DIDOPO	53	428.24	801.28	193.21	17.513	27.198	5.3	98.3
PEO/15% MCSW	53	507.69	931.31	199.07	11.687	29.388	6.01	96.2
PEO/10% DIDOPO/5% MCSW	49	394.74	670.58	190.94	0.22	30.691	4.49	89.6

**Table 4:** Three flame-retardant effects.

Sample	Flame Inhibition Effect (%)	Barrier Protective Effect (%)	Charring Effect (%)
PEO/15% DIDOPO	10.7	20.3	1.1
PEO/15% MCSW	3.5	10.1	3.2
PEO/10% DIDOPO/5% MCSW	−0.01	32.5	9.8

**Table 5:** Results of the TGA and DSC of the PEOs.

Sample	T_5%_	T_max_	Char Residue at 700 °C	T_m_	T_c_
	(°C)	(°C)	(wt.%)	(°C)	(°C)
PEO	273.96	401.5	-	58.15	42.07
PEO/15% DIDOPO	321.07	403.5	-	57.22	34.26
PEO/15% MCSW	295.45	400.9	2.31	57.00	37.03
PEO/10% DIDOPO/5% MCSW	344.51	403.3	7.82	58.88	34.17

**Table 6:** Elemental contents, as determined via EDS, of the char residues generated via cone calorimetry of the PEOs.

Sample		Elemental Content (wt.%)				
	C	N	O	P	S	Ca
PEO	10.4	4.42	85.18	--	--	--
PEO/15% DIDOPO	13.57	3.42	54.08	28.93	--	--
PEO/15% MCSW	3.94	2.18	43.57	--	21.24	29.08
PEO/10% DIDOPO/5% MCSW	6.54	2.23	33	13.68	8.82	26.97

## Data Availability

The original contributions presented in this study are included in the article. Further inquiries can be directed to the corresponding authors.
